# Influence of communication determinants on safety commitment in a high-risk workplace: a systematic literature review of four communication dimensions

**DOI:** 10.3389/fpubh.2023.1225995

**Published:** 2023-08-08

**Authors:** Jamil Zara, Shahrina Md Nordin, Ahmad Shahrul Nizam Isha

**Affiliations:** Center of Social Innovation, Department of Management and Humanities, Universiti Teknologi PETRONAS, Seri Iskandar, Malaysia

**Keywords:** safety communication, safety commitment, communication climate, communication satisfaction, occupational safety, high-risk workplace, occupational accidents, occupational injuries

## Abstract

Health, safety, and environment (HSE) are critical aspects of any industry, particularly in high-risk environments, such as the oil and gas industry. Continuous accident reports indicate the requirement for the effective implementation of safety rules, regulations, and practices. This systematic literature review examines the relationship between safety communication and safety commitment in high-risk workplaces, specifically focusing on the oil and gas industry. The review comprises 1,439 articles from 2004 to 2023, retrieved from the Scopus and Web of Science databases following the PRISMA comprehensive guidelines. This study considers safety communication, communication climate, and communication satisfaction to evaluate their influence on safety commitment under occupational health and safety. This study identifies safety commitment issues and their underlying factors, discussing measures for preventing and reducing accidents and incidents and highlighting preventive measures for future research. It also signifies the variables influencing accident and incident rates. The research underscores the importance of communication dimensions and the need for workers to possess adequate skills, knowledge, and attitudes regarding occupational safety and health procedures. Moreover, the study contributes to the industrial and academic domains by improving organizational safety commitment, promoting a safety culture, and developing effective communication strategies. Furthermore, practitioners may benefit from this comprehensive overview in developing, evaluating, and enhancing occupational safety.

## 1. Introduction

For decades, the oil and gas industry has been considered a highly effective industry for accelerating the world’s economies (1). Malaysia has Asia-Pacific’s third and fourth-highest gas and oil reserves, respectively (2). Therefore, the contribution from these industries accounts for approximately 20% of GDP in the Malaysian economy (3). Despite implemented safety measures, oil and gas operations are reportedly significantly challenging and dangerous globally, whether onshore or offshore (4), raising concerns regarding highly hazardous and risky processes and associated activities in the oil and gas industry. These incidents and perilous circumstances occur due to ineffective communication and negligence of defined rules, regulations, and commitment to safety (5). Though the deployment of rules, regulations, and technology has drastically improved safety measures, oil and gas industry incidents are still significantly higher than in other industries and can trigger a catastrophic impact, leading to a high rate of occupational injuries and accidents, as reported by numerous studies (6, 7). Therefore, ensuring a safe working environment is of utmost importance.

Workplace safety is associated with active communication to minimize the rate of accidents and improve safety in the workplace. It is also deduced that the antecedent of accidents is unsafe behavior, which is partly ascribed to the organization’s safety systems (8). According to International Labor Organization (ILO) global statistics reports, nearly 2.78 million fatalities are reported annually. Poor and inefficient safety systems, management practices, and human error are among the causes of the reported incidents. Such consequences are alarming and necessitate a focus on improving the workplace to reduce workplace accidents, injuries, and fatalities, which also accrue costs to the organization in terms of medical, health, death, equipment damage, production loss, and increased insurance costs (9–13).

Moreover, the Social Security Organization - SOCSO (2018–2019) has expressed dissatisfaction with the oil and gas sector’s Health, Safety, and Environment (HSE). The most viable indicator of workplace safety is the number of fatalities and injuries that have occurred; therefore, most companies endeavor to achieve ‘zero lost time injury’ (LTI). Despite such preferences to ensure workplace safety the oversight occurs, which lead towards hazards (14–16). These incidents are heavily interlinked with communication and human error, highlighting the importance of effective communication in the workplace to avoid incidents (17).

The Malaysian Occupational Safety and Health Act (OSHA) was passed on the 25th February 1994. It has significantly improved the health and safety of working environments in Malaysia and influenced oil and gas organizations to comply to avoid unpleasant incidents and near-misses (18–20). Despite the ILO and the Malaysian OSHA-1994, the frequency of incidents indicates that acceptable safety measures against risks are not being provided. These risks are associated with safety performance, which comparatively exists due to a lack of safety leadership, safety commitment, and safety communication (19, 21). A safe workplace is facilitated by the employees’ and employers’ strong support and commitment to safety. Numerous studies report ineffective communication to be a significant factor in triggering accidents (18, 19, 22, 23).

**Figure 1:**
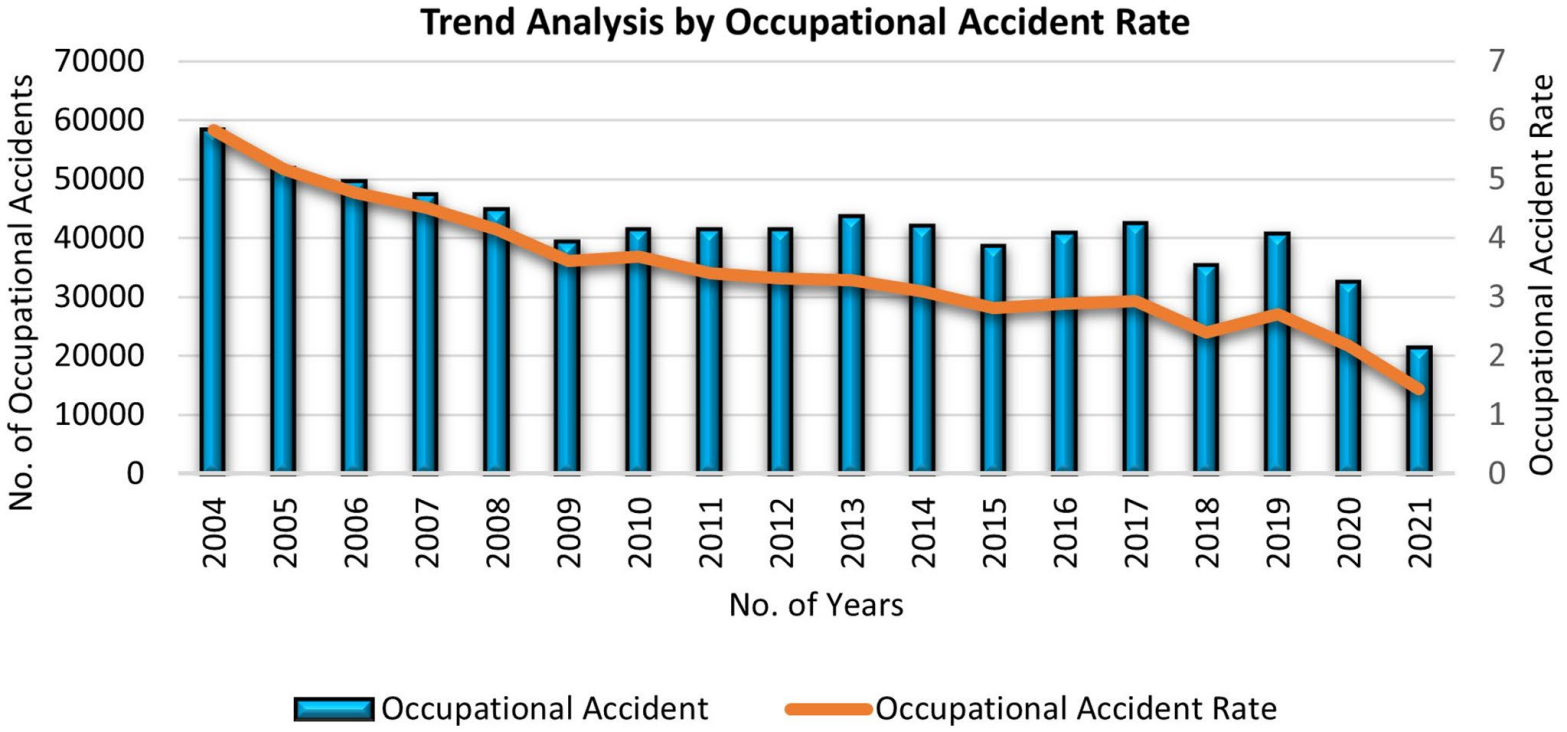
Trend analysis by occupational accident rate published by the Department of Statistics Malaysia for 2021 (24).

**Figure 2:**
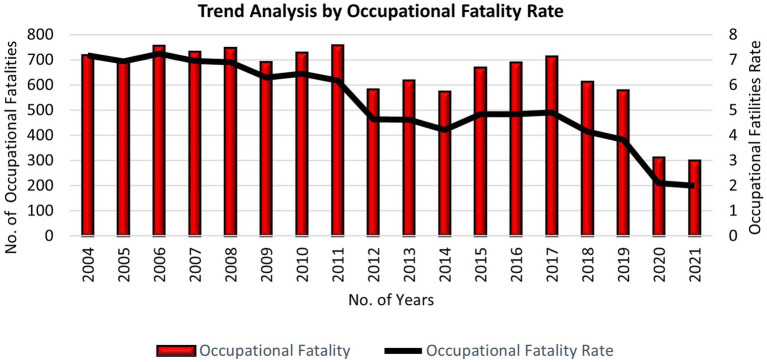
Trend analysis by occupational fatality rate published by the Department of Statistics Malaysia for 2021 (24).

**Figure 3:**
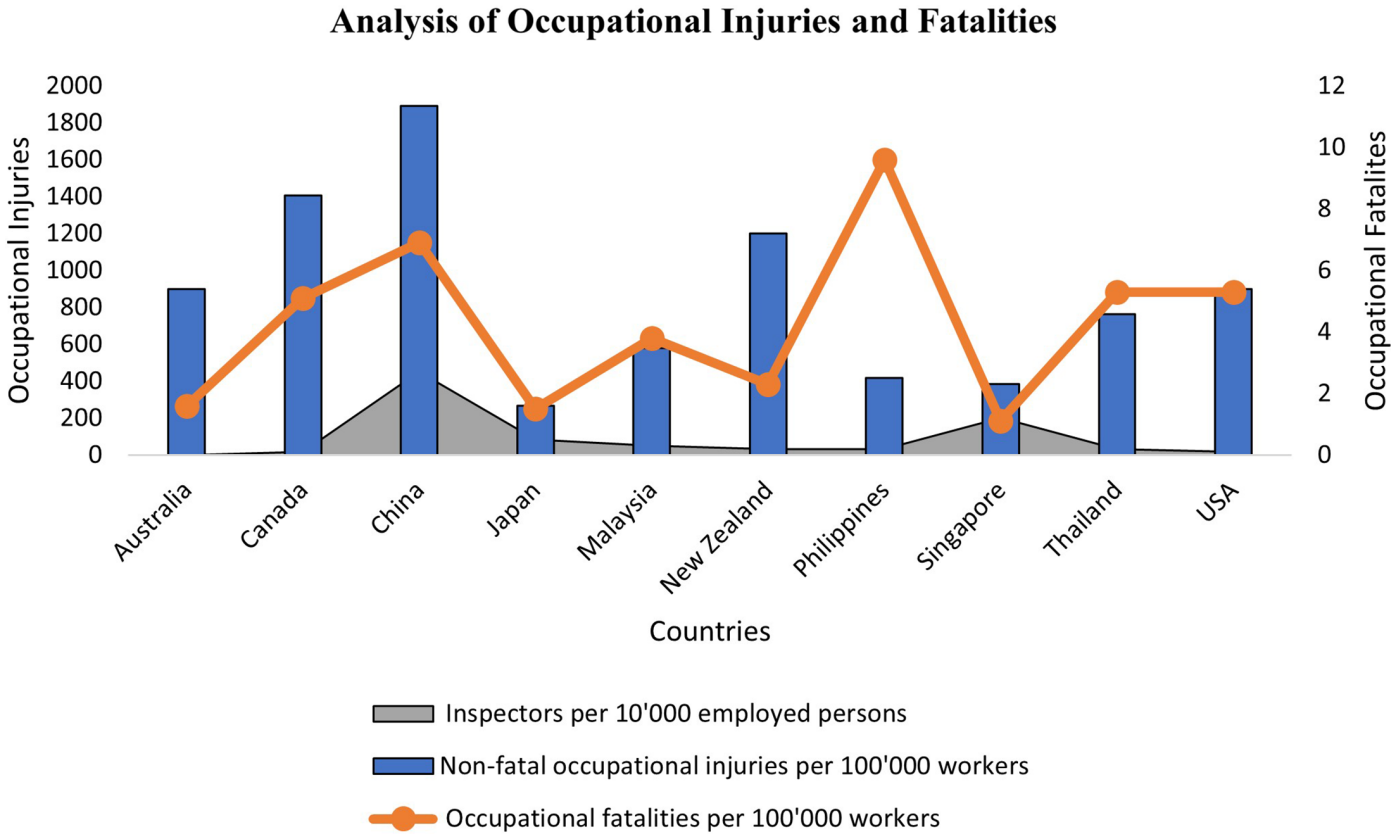
Comparative analysis of different countries by occupational injury and fatality rates (ILOSTAT).

Although the rate of accidents has decreased globally compared to previous years due to safety precautions and HSE implementation, including the technological advances of equipment and machinery, the human factor has significantly contributed to accidents in the workplace. Figures 1, 2 present a trend analysis of the occupational accident and fatality rate in Malaysia from 2004 to 2021, and Figure 3 presents a comparative analysis of the occupational injury and fatality rates of different countries. The statistical evidence indicates that approximately 80–90% of incidents are derived from human factors (25). These factors are not limited to human and machine contact but extend to various factors involving individuals, organizations, and working environments (26). Therefore, safety communication is considered a significant factor in reducing accidents caused by human factors and improving the workplace (27). However, the SOCSO produced the latest report claiming that in 2021, a total of 61,447 accident cases were reported, of which, 36,794 were industrial accidents, with almost 700 reported deaths in Malaysia alone. However, According to worldwide evidence, 2 million fatalities have been reported relating to accidents and diseases caused by the workplace. In total, 270 and 160 million occupational accidents and illnesses occur yearly, respectively (28, 29). Workplace safety remains challenging and it is needed to avoid accidents effectively and improve safety performances (30).

Unsafe working conditions generate serious health issues and affect employees’ well-being, negatively impacting their productivity and organizational performance (31). Human behavior is anticipated to be dynamic and complex (32); therefore, their capacity to understand, comply with, and perform according to the procedures and standards of safety contributes significantly to achieving success across high-risk industries (33–35). Organizations should consider effective communication and the efficient administration of OSHA practices to improve work performance in high-risk environments and promote positive working environments. Therefore, effective communication is of utmost importance to ensure positive working behavior (36).

However, communication barriers pose a significant challenge in promoting safety in an organization (37, 38); ensuring credibility, impact, and clarity remain vital to safety communication (39). Safety communication comprises a broad spectrum, from entry to board level, and is believed to be an essential factor connected with unsafe behavior (40). The unsafe behavior of employees may ultimately expose them to work-related accidents or injuries due to a lack of safety awareness. Therefore, safety communication also ensures employees’ behavior is monitored so that they are less likely to exhibit unsafe behavior (41, 42). Several barriers hinder effective communication, including lack of information, knowledge, and attention; the existence of prejudice; different perceptions; selective listening; and a lack of clear reporting of official information (16, 43). Therefore, effective communication is critical to ensure safe operations and avoid disasters at oil and gas plants.

There is a surfeit of existing research evidence supporting the theory that work-related safety behavior could be improved by effective communication, which directly correlates with the significant association between communication and safe behavior (8–10, 16, 27, 39, 40, 44–46). Some recent studies have investigated the relationship between commitment and communication to constitute the impact of safety communications, leader and worker roles, and organizational climate in the oil and gas industry (46, 47). Most studies have revealed that the safety communication and climate of an organization are crucial antecedents of safety commitment.

The existing studies have highlighted the communication issue, indicating numerous complexities in several communication domains (mentioned in Table 1). However, these studies have limited dimensions and highlight the issues instead of emphasizing the root causes. Henceforth, there is a need to further investigate, identify, and imply how the four dimensions of communication affect safety and assist in preventing disasters at high-risk workplaces to reduce the high rate of accidents, injuries, and fatalities causing physical and mental health, and investment loss.

**Table 1:** Analysis of themes and sub-themes for safety communication.

Reference and country	Safety communication				Occupational health and safety						Safety performance				Safety commitment			
	CC	CS	CM	CE	SP	SI	Sin	SR	S	SB	OA	OF	NM	LT	SM	SK	SC	SP
(7) Italy								x			x						x	
(25) UK	x			x											x			
(48) Malaysia				x		x	x		x		x	x		x			x	
(31) Iran					x									x				
(34) Australia						x	x		x		x	x	x	x	x			x
(49) Malaysia	x			x	x				x	x	x		x			x		x
(50) Malaysia				x	x	x			x		x			x		x	x	x
(51) Norway											x						x	x
(52) China				x	x			x				x			x		x	x
(53) Indonesia			x				x				x	x		x				
(54) Norway									x		x			x				
(55) USA				x	x											x		
(56) USA	x							x										x
(57) Australia				x											x			x
(58) Brazil	x		x															
(59) France		x			x												x	
(60) Malaysia	x			x						x						x		x
(61) Norway	x				x	x		x										x
(62) Malaysia	x		x															
(63) Egypt	x	x													x	x		
(64) Brunei						x		x				x		x				
(65) Indonesia	x			x	x										x		x	
(66) Nigeria	x																	
(67) Albania	x	x	x												x	x		x
(68) Columbia	x	x	x							x								
(69) Australia	x			x	x										x		x	
(70) Croatia	x		x	x	x										x			
(71) Malaysia						x	x				x	x	x	x		x		x
(72) Israel				x		x					x				x	x	x	
(73) Malaysia										x	x	x		x	x		x	x
(74) Ghana				x	x	x					x			x	x	x		x
(75) China					x		x		x		x			x	x	x	x	
(76) UAE					x	x		x				x		x		x	x	
(77) Norway				x			x	x				x						
(78) Singapore						x	x					x	x					x
(79) Bahrain												x		x		x		
(80) Iran										x								
(81) USA	x			x				x								x		
(82) UK								x		x	x							

Safety Communication	Occupational Health and Safety	Safety Outcome	Safety Commitment
CC: Communication Climate	SP: Safety Policy	OA: Occupational Accidents	SM: Safety Motivation
CS.: Communication Satisfaction	SI: Safety Issues	OF: Occupational Fatal Accidents	SK: Safety Knowledge
CM: Communication Mechanism	Sin: Safety Indicator	NM: Near Misses	SC: Safety Compliance
CE.: Communication Effectiveness	SR: Safety Rules and Regulation	LT: Lost Time Injuries	SP: Safety Participation
	S: Strategies		
	SB: Safety Benchmark		

Therefore, the current study presents a systematic literature review based on four communication dimensions after examining the past studies. The proposed study focuses on highlighting safety issues and identifying communication gaps in high-risk workplace environments, especially in the oil and gas industry. Furthermore, this study also investigates and formulates research questions regarding how safety communication, communication satisfaction, and communication climate influence safety commitment. The deductions of these research questions will assist in avoiding disasters in a high-risk workplace environment, specifically focusing on oil and gas processing plants in Malaysia.

## 2. Methodology

The methodology adopted in this research is based on a systematic review to evaluate the literature regarding safety communication, communication climate, communication satisfaction, and safety commitment to explore the findings and highlight existing research gaps for future studies in the context of occupational health and safety. As per the Preferred Reporting Item for Systematic Reviews (PRISMA) 2020, systematic literature is based on explicit, systematic procedures for gathering and synthesizing data from research that covers a specific area of study. Though most literature reviews are part of a broader perspective of study, they can be a stand-alone piece of work (83).

This type of study combines the results of other investigations to create a logical and comprehensive argument for a specific research issue (84). To define a future research agenda based on the existing gaps, a review article’s main objective is to critically analyze the literature in a particular research area, subject, or field, identifying significant theories, key constructs, and empirical methods and setting unaddressed research questions (85). Systematic research reviews have gained popularity in research trends because of the data and information that they can provide. In conducting and directing this systematic review, the protocols suggested by Page et al. (84) suggested protocols were used as a reference. A five-step strategy, which consists of the following points, has been proposed (86).

(i)Framing review questions
(ii) Identifying appropriate works
(iii) Evaluating the quality of studies
(iv) Synthesizing the evidence
(v) Interpreting the results

PRISMA guides comprehensive and systematic research regarding the term “safety communication” and its influence on safety commitment, safety performance, and outcomes. This study follows the main three simple review steps:

(i) Developing review questions
(ii) Identifying appropriate literature
(iii) Synthesizing the relevant forms of works

The current study was conducted from November 2022 to March 2023. This study includes three primary aspects, namely, the oil and gas industry (population), the practice of safety management (context), and safety metrics (interest), to achieve the study’s objective and answer the research questions.

### 2.1. Resources

This study uses two major databases to conduct a systematic review, namely, Scopus and the Web of Science, due to their strength and inclusion of more than 334 disciplines and areas of research, including occupational health and safety studies. However, it should be kept in mind that no database, not even Scopus or Web of Science, is comprehensive and flawless.

### 2.2. Research question formulation

The formulation of the research question is considered the first step in a systematic review; it should be formulated and stated clearly before the study is started (86). PICO, which stands for Population, Intervention, Comparison, and Outcome, can often be used to identify the key components that must be addressed to fully formulate the review question. The main objective of this review is to examine the impact of safety communication on safety commitment in high-risk work environments within the oil and gas industry. Specifically, the study focuses on three primary aspects: the population involved (oil and gas industry), the intervention being studied (safety communication practices), and the context and outcome of the research (influence on safety commitment). The comparison aspect is not included in this study, as the main emphasis is on understanding how safety communication practices influence safety commitment in the high-risk workplace setting (87). Therefore, the following three research questions were devised for the purpose of this study:

RQ1: How does safety communication impact safety commitment in a high-risk work environment?

RQ2: How does communication satisfaction influence safety commitment in high-risk work environments?

RQ3: How does communication climate impact safety commitment in high-risk work environments?

### 2.3. Systematic searching strategies

Based on PRISMA, there are three main methods in the systematic searching strategy process, namely, identification, screening, and eligibility (83, 88).

#### 2.3.1. Identification

Identification is a search method that uses the study’s primary keywords, i.e., communication, occupational health, and safety, which were developed based on the research question (89). The search process used synonyms, associated keywords, and variants to give users more options for choosing databases when searching for further relevant articles for the review. The identification procedures were based on prior research, keywords proposed by guidelines, and keywords suggested by experts. In this study, entire search strings were constructed using an enriched existing term from the Scopus and Web of Science databases. A total of 1,439 articles were found during a search of these two databases from Dec 2022 to March 2023 that was limited to published papers from 2004 to 2023. Table 2 provides details about the databases and search strings considered.

**Table 2:** Search strings followed for literature extraction from the Web of Science and Scopus.

Data base	Search string
Web of Science	TOPIC: (“safety communication” OR “effective safety communication” OR “effectiveness of safety communication” OR “impact of safety communication” OR “effect of safety communication” OR “occupational safety and health” OR “OHS performance” OR “safety indicators outcome” OR “leading indicator” OR “lagging indicator”) Refined by: TOPIC (“effective communication management” OR “communication management practice” OR “safety commitment” OR “safety programs” OR “communication climate” OR “communication satisfaction”) AND FINAL TOPIC: (“industries” OR “oil” OR “gas” OR “petrochemical” OR “oil and gas sector” OR “high-risk work environment” OR “hazardous workplace”).
Scopus	TITLE-ABS-KEY ((“safety communication” OR “effective safety communication” OR “effectiveness of safety communication” OR “impact of safety communication” OR “effect of safety communication” OR “occupational safety and health” OR “OHS performance” OR “safety indicators outcome” OR “leading indicator” OR “lagging indicator”) AND (“effective communication management” OR “communication management practice” OR “safety commitment” OR “safety programs” OR “communication climate” OR “communication satisfaction”) AND (“industries” OR “oil” OR “gas” OR “petrochemical” OR “oil and gas sector” OR “high-risk work environment” OR “hazardous workplace”)).

#### 2.3.2. Screening

The objective of the initial screening was to remove duplicate articles. A total of 17 duplicate articles were removed from the chosen articles. According to the numerous inclusion and exclusion criteria developed by the researchers, 1,088 publications were removed during the first stage, and 334 articles were reviewed during the second stage. The researcher decided to only focus on journal sources and article document types (research articles) because they act as the main sources of empirical data for determining the first criterion, which was the type of literature. As a result, this study did not include any conference papers, book chapters, reviews, conference reviews, notes, abstract reports, business pieces, brief surveys, retracted works, conference proceedings, trade journals, book series, books, or book chapters. Second, all non-English publications were removed from the search, and only English-language articles were prioritized to avoid misinterpretation and translation issues. Furthermore, 20 years (between 2004 and 2023) was selected as an appropriate time frame for tracking the development of research and related publications. These standards led to the elimination of 1,088 articles in total to achieve the objective of the study. Table 3 outlines the criteria for inclusion and exclusion of articles.

**Table 3:** Criteria for articles inclusion and exclusion.

Criteria	Inclusion	Exclusion
Timeline of publication	2004- March 2023	2003 and before
Types of documents	Articles (research on empirical data and review)	Conference papers, book chapters, book series, conference reviews, books, short surveys, notes, reports, etc.
Language	English	Non-English
Discipline of the study	Safety communication practice in industries Measurement of safety indicators Safety outcomes	Research methodology/Process system. Not relevant in terms of safety communication. Not related to the high-risk workplace environment.

#### 2.3.3. Eligibility

Eligibility involved individually reviewing the relevant articles to ensure that all the remaining research articles met the research criteria after the screening process. This process was completed by reviewing the article’s title, abstracts, and keywords, which were used for literature coding. Based on the unnecessary or irrelevant information mentioned in the title, abstract, and keywords, the article was analyzed to aid in coding. The coding process identifies study methods and findings portions. During this process, the following information was collected and included in the database:

(i) Title of the paper
(ii) Publication year of each paper
(iii) Region or country (this information is regarding the location of the research article and is not related to the author’s origin)
(iv) Research area and field
(v) Population of the research (profession)
(vi) Safety communication context and concept

**Figure 4:**
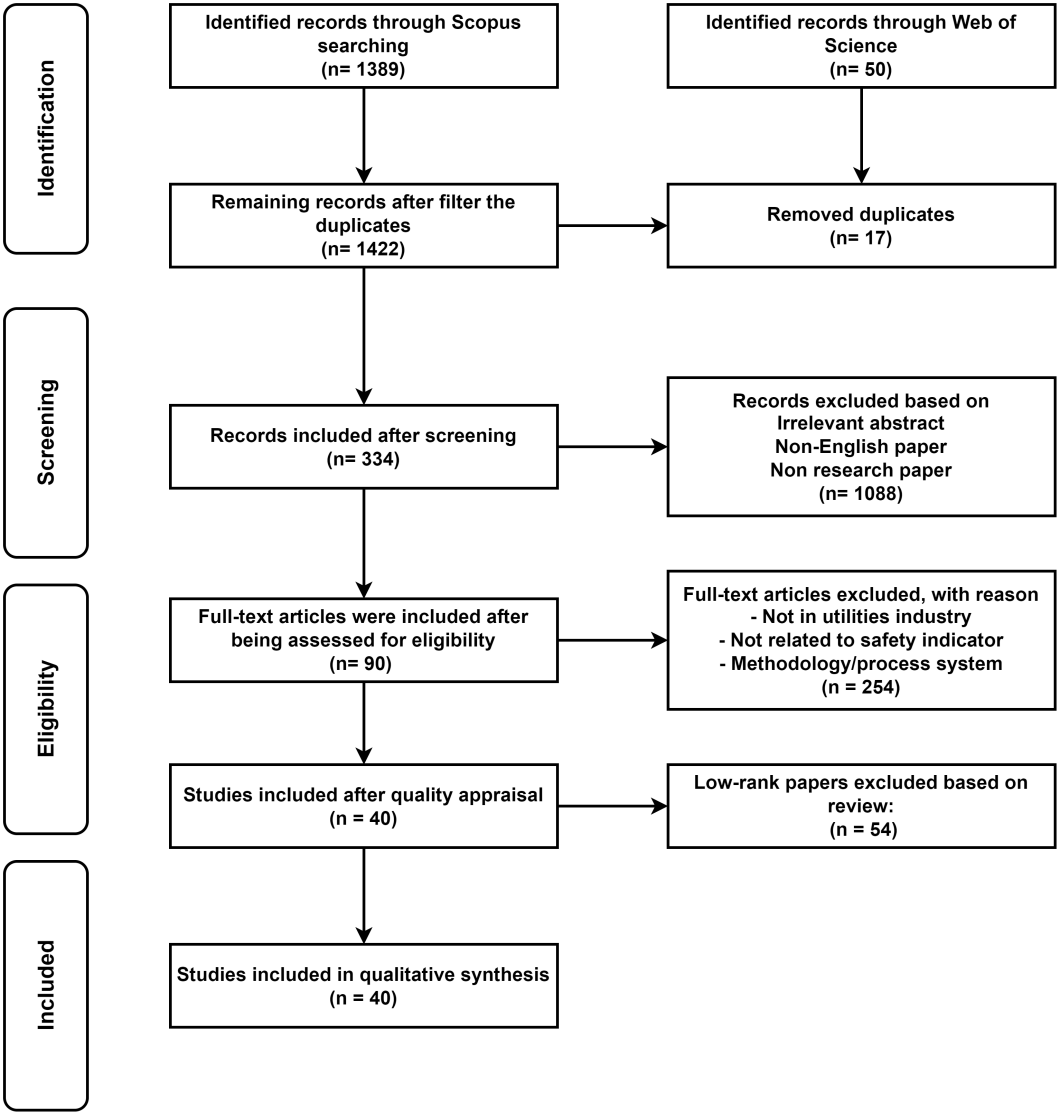
Systematic literature review process following PRISMA guidelines.

A standard procedure was adopted based on PRISMA 2020 (presented in Figure 4) to reduce the chances of biases and to ensure the quality of the literature review. The exclusion criteria included studies conducted in non-high-risk work industries, not related to safety communication, irrelevant to the health and safety field, and published as a book chapter. After the assessment of quality, a total of 254 articles were removed and 90 were selected.

#### 2.3.4. Data abstraction and analysis

This study used an integrative review method, which analyses and synthesizes different research methodologies (qualitative, quantitative, and mixed methods) together, to reduce biases and ensure all data can be gathered by converting one kind of data into the other, i.e., qualitizing quantitative data or quantitating qualitative data (90). The data were acquired based on qualitative analysis in this study. The process of developing relevant themes and sub-themes was carried out based on thematic analysis. Data collection was the first step in the theme creation process. To find statements or information that addressed the study questions, the authors carefully read through a group of 40 publications during this phase. The authors employed a coding approach to create meaningful groupings based on the nature of the data in the second stage after that examination. In other words, by discovering themes, concepts, or ideas for more connected and related data, the second stage turned raw data into usable data (91).

As a result, the authors noted any discrepancies in the themes that emerged. The authors also reviewed the results to address any disparities in the theme-generating process. The developed themes and sub-themes were then modified as necessary to maintain consistency.

#### 2.3.5. Inclusion and exclusion criteria

After setting the research questions, we set the limitations for article selection based on the inclusion and exclusion criteria. The inclusion and exclusion criteria for the study were developed on the principles of systematic review protocols and PRISMA 2020 guidelines. These criteria are described in the following section.

##### 2.3.5.1. Inclusion criteria

The inclusion criteria for selecting articles in our study included articles published between 2004 and March 2023. We considered articles based on empirical data and review studies, ensuring a comprehensive examination of the topic. The language requirement was English to facilitate a thorough understanding and analysis of the content. Our focus was on safety communication practices within various industries, examining how organizations communicate and promote safety within their workforce. Furthermore, we prioritized articles that investigate the measurement of safety indicators, allowing us to assess the effectiveness of these practices. Finally, we considered articles that explore the impact of safety communication on safety outcomes, as this provides valuable insights into the overall effectiveness of these practices. By adhering to these inclusion criteria, we aimed to provide a comprehensive and insightful analysis of safety communication in industries.

Moreover, the inclusion criteria of the study also included quality appraisal, where two experts with 15–20 years of experience as an auditor from health and safety backgrounds were selected. The selected articles were sent to the experts for quality assessment to ensure the high quality of the content. The remaining publications were divided into three categories: high, medium, and low. Articles in the high and medium groups were selected for review. The papers were classified when both experts were satisfied with the quality and ranking. This methodology produced 12 with a high ranking, 28 with a medium ranking, and 50 that were considered low-ranking articles. As a result, low-ranking articles were excluded, and the remaining 40 articles were considered suitable for further examination.

##### 2.3.5.2. Exclusion criteria

The exclusion criteria for our study ensured that we focused on the most relevant and rigorous research in the field of safety communication. We excluded articles published before 2003 to ensure we captured the most recent advancements and insights in the field. Additionally, we excluded conference papers, book chapters, book series, conference reviews, books, short surveys, notes, reports, and similar publications to maintain a high standard of scholarly research. Non-English articles were also excluded to ensure consistency in our analysis and interpretation. We excluded articles that primarily focus on research methodology or process systems rather than the specific topic of safety communication. We also excluded the articles that were selected as low-ranking after quality appraisal.

Furthermore, the content of each article was then carefully assessed to determine its relevance to the topic of safety communication in industries, measurement of safety indicators, and safety outcomes. Articles that did not address these specific areas or were not related to the high-risk workplace environment were excluded from further analysis. By applying these exclusion criteria, we aimed to maintain a focused and comprehensive analysis of safety communication in the context of high-risk workplaces.

## 3. Results

The literature review resulted in four main themes and 18 sub-themes related to factors impacting safety communication and commitment in high-risk work environments. As shown in Table 1, the four main themes comprise safety communication (four sub-themes), occupational safety and health (six sub-themes), safety commitment (four sub-themes), and safety performance (four sub-themes). The results proposed a systematic analysis of the factors impacting safety communication on safety commitment in a high-risk work environment.

### 3.1. Background and findings of the reviewed studies

An analysis led to the development of a total of four main themes, namely, safety communication, occupational health and safety management system, safety commitment, and safety outcomes. Following this categorization, the study procedures were followed for each of the themes that had been created, developing themes, ideas, or thoughts that were related to one another within the main subject as a sub-theme. This process resulted in 16 sub-themes. The data were categorized into four main indicators. There were four sub-themes in safety communication (communication climate, communication satisfaction, communication mechanism, and communication effectiveness), four in occupational health and safety management system (organizational policy, strategy, indicator, continual improvement), four in safety outcome (occupational accidents, occupational fatal accidents, near misses, and lost time injuries), and four in safety commitment (safety motivation, safety knowledge, safety compliance, and safety participation). The main themes and subthemes are presented in Table 1.

### 3.2. Years of publishing of selected articles

**Figure 5:**
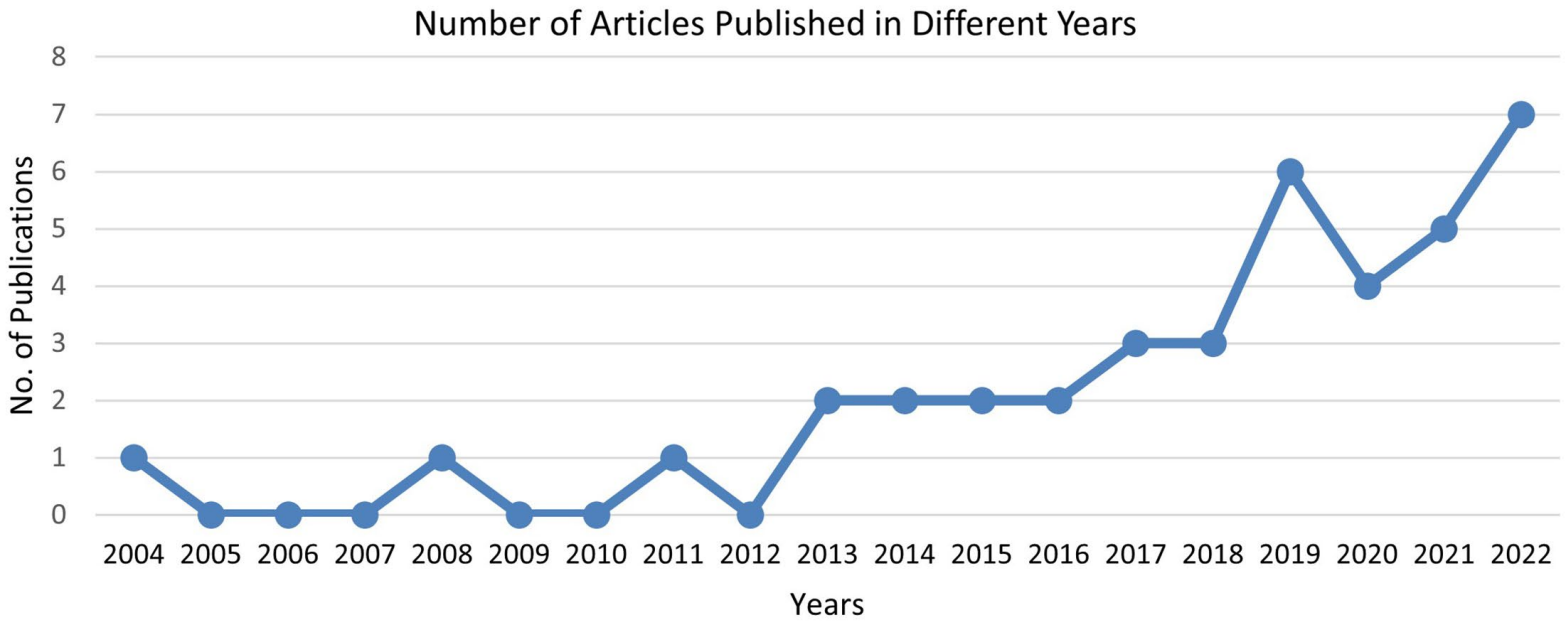
Number of articles published in different years on safety communication.

According to the literature, most of the articles on safety communication in the oil and gas industry were published in 2022, with seven articles (15%) and six articles (15%) respectively published in 2019–2021. The distribution of articles fluctuated during the study period, with only one article being published in 2004 and 2008, and the number of publications increased in 2013. However, this study was conducted from December 2022 to March 2023 and the researchers were not able to include articles that were published during these months. None of the articles were published in 2009–2012, except one, which was published in 2011. The fluctuation in publications indicates that researchers are directing their attention towards positive actions and proactively addressing safety communication issues to potentially minimize highlighting of occupational health and safety (OHS) accidents and injuries. Figure 5 presents the number of publications in the literature related to safety communication in different years.

### 3.3. Publication by origin/country

**Figure 6:**
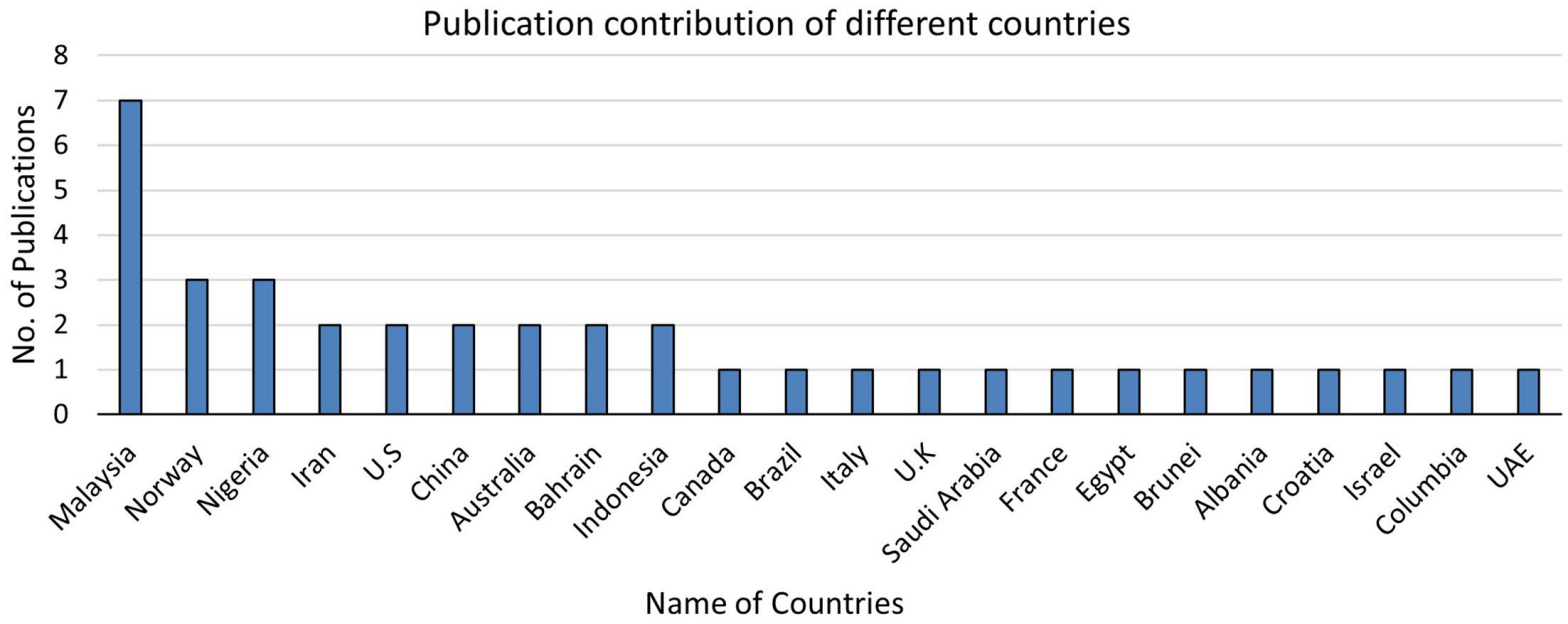
Publication contribution of different countries.

The countries where the highest number of studies were conducted were Malaysia, with seven articles (18%); Nigeria, with three articles (8%); and Norway, with three articles (8%), followed by China, Iran, the U.S., Australia, Bahrain, and Indonesia, each with two articles (5%). Most of the countries published one article only, including Brazil, Italy, the UK, Saudi Arabia, France, Egypt, Brunei, Albania, Israel, Croatia, Columbia, Singapore, and the UAE. Figure 6 presents a glimpse of the published articles in different countries.

### 3.4. Publication by field and methods

**Figure 7:**
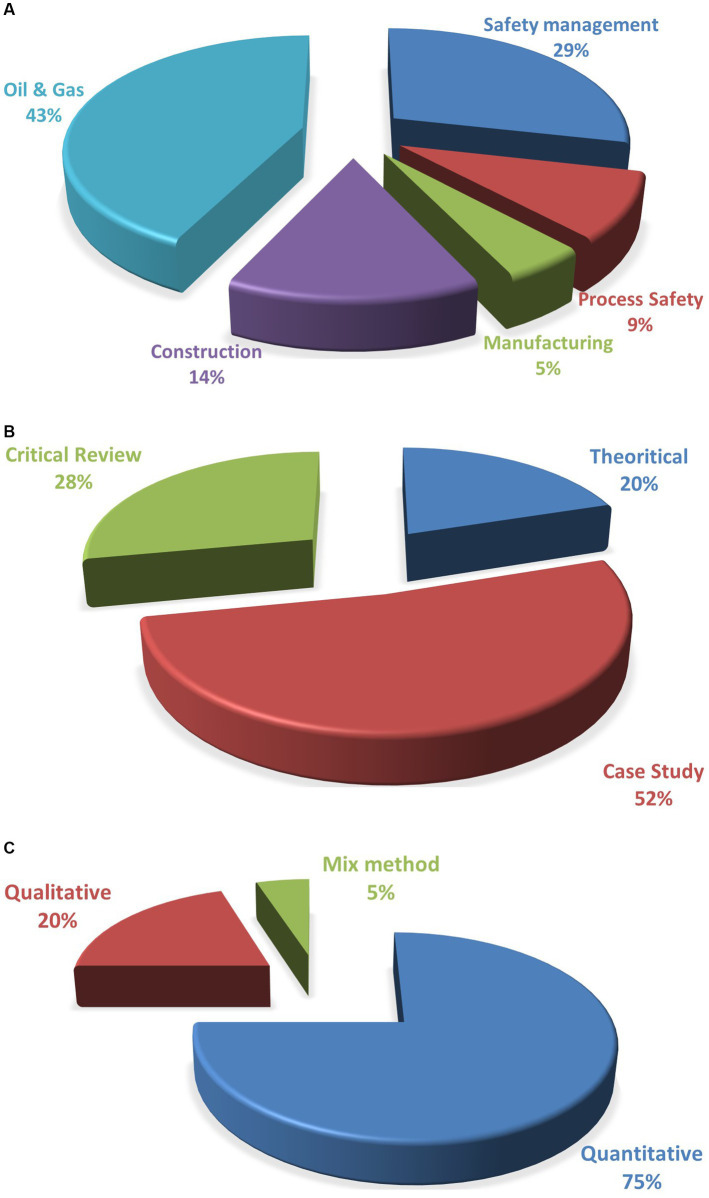
**(A)** Distribution of publications with reference to study areas. **(B)** Distribution of publications with reference to study methodologies. **(C)** Distribution of publications with reference to study category methods.

In 1970, the U.S. Congress enacted the U.S. Occupational Safety and Health Act of 1970 (OSHA) in the United States. Since then, governments and organizations have put a lot of effort into the development of OSHA around the world. The implementation of OSHA is broad and has diversified into all industries and various job roles and positions. As a result of categorizing the relevant publications, we can see that the studies involving safety competencies in OHS were conducted in five main fields of work, as shown in Figure 7. There were 14 research works conducted in the safety management field, mostly in Malaysia. The development of safety communication research in the high-risk work environment field shows the importance of equipping the young workforce with core competencies before they join the occupational world. The remaining studies were conducted in other fields of work, namely, construction, process safety, safety management, manufacturing, and oil and gas. These four areas were the main implementation of OSHA in most regions. The oil and gas, construction, and manufacturing fields are old industries that are known for their high-risk tasks involving workers and machines. For the process safety field, the OHS risk is also high because this field consists of the chemical and petrochemical industry, which needs a strong, competent workforce. Most of the people who participated in these studies were members of safety management teams, workers, and construction supervisors. Hence, most of the studies applied a quantitative approach, accounting for 75%, while qualitative studies accounted for 20%, and a mixed method approach was adopted by only 5% of studies. It is worth mentioning that the most prevalent methodologies used in these studies are case study, accounting for 52%; critical review, accounting for 28%; and theoretical, accounting for 20%.

### 3.5. Themes and sub-themes of the study

This section discusses the four proposed main themes, namely, safety communication, occupational safety and health, safety performance, and safety commitment, which, between them, have 18 sub-themes (see Table 1).

#### 3.5.1. Safety communication

The safety of workers is crucial for all high-risk organizations, including in the oil and gas industry. One of the most critical factors associated with unsafe behavior is safety communication. “Safety communication is an appropriate knowledge exchange concerning internal safety matters. It enhances the efficiency of the safety management system.” An effective form of communication would help ensure safe workplace behavior. Safety communication guides workers to reduce unsafe incidents, strengthen their motivation, and commit themselves to safety in the workplace. Safety communication procedures also assist workers in improving safety practices in the workplace. Several studies have reported that a lack of safety communication is a significant factor in creating conflict and confusion regarding rules and procedures among workers in an organization (92). In this study, 23 articles (57%) from past studies were found to focus on safety communication, specifically in related sub-dimensions, impacting organizational safety performance and reducing accident and injury rates. Most highlighted variables that is communication climate and effective communication were the most prevalent and relevant approaches in this context, being the focus of16 papers (40%), followed by communication mechanism, which was the focus of 7 papers (17%), and communication satisfaction, as the focus of 4 (10%).

##### 3.5.1.1. Communication climate

The communication climate, in terms of organizational perspective, can be defined as “a relatively enduring quality of the organizational environment which is experienced by its members that could encourage or discourage the communication process” (93). The organizational communication climate plays a pivotal role in the success of an organization due to the relationship it creates between leaders and members. The effectiveness of communication between leaders and members nurtures the organization and it significantly impacts the act of following safety measures.

The communication climate is dominant and essential to a workplace as it contributes to the efficiency and success of an organization. The organizational communication climate influences the workplace atmosphere either by encouraging or discouraging both horizontal and vertical communication modes across all levels of employees. Supportive environments motivate effective participation, prompt and healthy exchange of information, and productive conflict resolution. An effective communication climate needs active and efficient management of conflicts (60, 93–96). Moreover, the internal climate positively impacts the correlation of organization leaders with other organization members. A study by (56), conducted in the United States, revealed that supportive communication from senior management with employees encourages symmetrical communication in the workplace; hence, balanced communication is required to create and promote the appropriate communication mechanism in the organizational climate.

Additionally, positive internal communication is crucial in public relations; organizations gain positive outcomes, such as workers’ involvement, commitment, and performance (56). Symmetrical internal communication is significant for an organization as it has greater potential to enhance personnel creativity. Researchers (97) have argued that workers’ perspectives regarding a symmetrical communication climate and systems are highly influenced by transformational leadership behavior (e.g., trained leadership style, authentic leadership). A symmetrical communication climate, two-way communication, and an open environment can foster and encourage personnel’s active communication within their organization. It can be concluded that these factors influence their feedback-seeking behavior FSB (78, 98). Pragmatic research has shown that symmetrical internal communication is a contributing factor in achieving organizational visions and goals by involving individuals from different sectors and improving results of individuals to follow the safety precautions and develop sense of belonging, such as corporate empowerment identification, trust, and the relationship between employee and organization (56, 99).

##### 3.5.1.2. Effective communication

One-way communication and feedback are not enough for effective communication (100). Morning meetings, toolbox discussions, safety walks, and workshops are thought to stimulate conversations and contribute to openness and trust within an organization. Employee communication is critical for carrying out safety procedures. As a result, the organization should adopt safety measures, such as enabling two-way communication with employees, especially within multicultural workforces (101). This entails creating a safety information system that collects, analyzes, and disseminates data from accidents and near misses, as well as performing proactive checks on the system’s vital signs on a regular basis (102). Furthermore, organizational management may be successful to communicate the effective rules and regulations to their employees to reduce accident and injuries (103). As a result, techniques and resources, such as leading indicators and flexible communication channels, are needed to facilitate the efficient exchange of information among individuals (100). Accidents and unsafe conditions are expected to be reported on a regular basis, resulting in early problem resolution before employees are harmed, and results are expected to be communicated on a regular basis (104). However, when combined with frequent interaction with safety communication, employees’ dependability and positive outcomes should increase rather than decrease (105).

##### 3.5.1.3. Communication mechanism

Communication is considered essential for giving feedback on how employees perform their tasks and for providing knowledge and information according to the organization’s vision regarding reducing safety issues and hazards. It motivates, provides a direction, and illuminates possibilities for improvement and collective growth (106, 107). Verbal communication is commonly used for the delivery the safety information. According to leaders, it is of utmost importance to provide employees with clear information regarding the need for safety rules and procedures and find the reasons behind why certain rules and regulations usually face conflict. Through the proper channel of communication, employees receive instruction regarding safety procedures and standards, and they ensure they are understood before beginning operational tasks (108). Communication mechanisms are key tools used by management to deliver the objectives and visions of an organization.

Organizational communication commonly shares information about function and relationship structure among workers. This flow of information has limitations, such as the horizontal and vertical flow of information (109, 110) stated that organizational communication significantly affects employees’ performance. It improves their efficacy and effectiveness, in which top-down communication could impact capabilities and roles and workforce in decision-making, enhance productivity, and motivate workers to demonstrate more competent and effective performance. However, bottom-up communication assists in maintaining a supportive and vertical structure for awareness, thus empowering a balanced work environment to achieve worker efficacy and effectiveness. In addition, organizational communication is intended to encourage informal communication and bottom-up efforts. The importance of consistent and up-to-date communication and effective tools for putting plans into action has been emphasized. Safety briefings, newsletters, information displays, films, safety days and events, monthly safety themes, and mobile applications are among the media and methods used to achieve safety communication and reduce accident rates (100). Furthermore, through monthly lessons learned and documented, the organization may communicate to its employees summaries of all accidents and process failures (including all first aid and near-miss events) (111). A poor safety and health culture, on the other hand, may lead to weaknesses because poor communication causes difficulties for employees in the workplace (101).

##### 3.5.1.4. Communication satisfaction

Communication satisfaction encompasses personal and individual satisfaction with multiple aspects of communication in interpersonal, group, and organizational contexts (112, 113). As per (70), communication satisfaction is generally deemed an affective reaction achieved by fulfilling an expectation during an exchange of information and implies a satisfying experience. Previously, communication satisfaction has been considered a unidimensional construct; however, various researchers (114, 115) have proven that it has multiple dimensions.

The theory of communication satisfaction was initially proposed to describe the relationship between communication and job satisfaction. Considered the most successful research framework for organizational communication (70, 116). Communication satisfaction includes the following constructs: communication climate, communication with management and subordinates, corporate contribution, horizontal and informal communication, media quality, organizational’ perceptions, and individual feedback (117). The organization should share all of its visions and values clearly with employees as it makes them feel more valued and motivated toward organizational commitment.

Organizational communication has multiple dimensions, including informational and relational dimensions (113, 118, 119). Informational communication satisfaction refers to satisfaction in an organizational context and exchanging information with team members, supervisors, and others concerned. Informational communication satisfaction relies on communication climate, managerial perceptions, and organizational integration. Relational communication satisfaction highlights satisfaction with the relationship between supervisors, subordinates, and all other organizational members in the workplace. It also refers to employees’ and supervisors’ perceptions and attitudes among corporate members (113).

#### 3.5.2. Occupational safety and health

The objective of OHS management is to provide and maintain a standard procedure for improving safety performance to prevent workplace hazards and injuries. It is a systematic mechanism to determine which methods are required to be implemented. Unsafe working conditions generate serious health issues and affect employees’ well-being, negatively impacting their productivity and organizational performance (31). Human behavior is anticipated to be dynamic and complex (32); therefore, their capacity to understand, comply, and perform according to the procedures and standards of safety contributes significantly to achieving success across high-risk industries (35, 120). Organizations should consider effective communication and efficient administration of OSHA practices to improve work performance in high-risk environments and promote positive working environments. Therefore, effective communication is of utmost importance to ensure positive working behavior (36). In total, 14 articles (35%) were based on occupational health and safety management system, specifically through effective safety communication, 10 (25%) articles were published on safety issues, 7 articles focused on safety indicators (17.5%), 6 papers highlighted the benchmark in the context of high-risk work environment (15%), organizational strategy was mentioned in 6 studies (15%), and the topic of safety rules and regulation was addressed in 10 articles (25%).

##### 3.5.2.1. Safety policy

A control component that prevents accidents from occurring is always present in organizations’ senior management, such as a safety policy and goals (103). According to organization management, companies follow a consistent safety policy throughout their operations. The importance of safety-related policies and programs is understandable because such rules and procedures typically provide immediate and visible proof of management’s commitment to safety. Organizational safety policies that are ignored or regularly violated send an unclear message about the importance of safety. It is also worth noting that most organizational policies include all aspects of the occupational safety and health management system, in addition to enforcement and discipline (102, 108). It is necessary to monitor and evaluate operational hazards by implementing relevant safety policies to protect and safeguard personnel and proactively prevent risks and accidents in the workplace. Safety indicators play a significant role in decreasing hazards and accident prevention. Safety indicators are used as operational variables in the oil and gas industry to monitor safety performance (35, 119, 120).

In Malaysia’s oil and gas industry, safety policies used to measure and monitor offshore processes are derived from generic-specific safety indicators published by the American Petroleum Institute and the International Association of Oil and Gas Producers (IAOG). The oil and gas sector remains a high-risk work environment with a high hazard and accident rate. There is growing concern that safety policies are not implemented correctly, resulting in fatalities, injuries, and incidents in the workplace (74, 121). A healthy and safe work environment requires safety policies and procedures. Due to the lack of appropriate safety policies, personnel experience safety issues, such as mental and physical disability, danger to life, property damage, and negative impacts on productivity and other colleagues (122). The significant components that cause these accidents are a lack of communication, negligence, and shortcomings in ensuring occupational health and safety in the workplace. Table 4 provides a description of different safety policies implemented in different countries.

**Table 4:** Description of different safety policies implemented in different countries.

References	Country	Safety Policies	Description
(123)	South Africa	OSHA	The safety policy of South Africa is adopted from the Occupational Health and Safety Act (OSHA). Its prime objectives and vision are to provide occupational safety for employees and health and safety for those who operate machinery and plant; to develop an advisory council for safety and health in the workplace.
(2)	Malaysia	HSE 2006	The Malaysian Oil and Gas industry adopts the HSE 2006 safety policy. That comprises a wide range of safety indicators that measure causes of accidents/ incidents or injuries and take responsibility for preventing accidents. Such accidents include but are not limited to fire, burnout, explosion, oil spills, gas release, or other conditions, which could result in illness or injury to personnel.
(124)	Indonesia	Regulation 79–2014	The Director-General of Oil and Gas (DGOG) monitors and implements policies and objectives associated with health and safety issues. Indonesia adopts safety policies from Government Regulation 79 of 2014 for the oil and gas sector. The government has stipulated that energy policy is to be implemented from 2014 to 2050.
(125)	Saudi Arab	OSHA	Saudi Arabia adopts the occupational safety policy from US OSHA standards and the American Society of Safety Engineers (ASSE). In the Jeddah refinery, multiple departments are responsible for maintaining employees’ health and safety, such as the Loss Prevent Department (LPD), Environment Protection Department (EPD), and the Occupational Medical Department (OMD). These all are working under Saudi Aramco Medical Services Organization.
(126)	U.K., Brazil, Mexico, and Australia	Regulation 2005	Brazil implements three safety policies, Safety Case, Barrier Management, and Research and Development, to maintain and establish a healthy and safe work environment for their employees in oil and gas organizations. The safety case approach is adopted from the UK’s offshore installation Regulation 2005. Under the safety case, operators are responsible for predicting safety issues, such as burns, escape, and fire risk, and fulfilling safety requirements at the workplace.

##### 3.5.2.2. Safety issues

The oil and gas industry is the most hazardous in the world. Even though operational safety procedures and standards with various effective programs are already implemented in Malaysia, incidents, hazards, and injuries still occur (125, 127). The high-risk work activities related to the oil and gas sector and minor incidents that become natural disasters make safety issues crucial in this field. Hazards in the oil and gas sector are always a reason for safety measures. It was identified that personnel in high-risk work environments suffer from work-related safety issues, including injuries, such as cuts, burns, contusions, and lacerations of the legs, hands, fingers, and eyes (128). The reasons for the occurrence of catastrophic events include poor infrastructure, lack of funding for safety systems, unqualified occupational health professionals, inappropriate occupational hazards and injury monitoring and evaluating mechanisms, and a lack of available data regarding health and safety; these are the most common safety issues in oil and gas industry (129). Along with these safety issues, employees face the most hazardous accidents and injuries that occur in the workplace. Table 5 provides a description of safety issues experienced in the oil and gas sector worldwide.

**Table 5:** A brief description of safety issues experienced in the oil and gas sector worldwide.

References	Year	Country	Safety issues
(2)	2017	Malaysia	The process of safety issues includes a set of various reasons; leakage and spillage, fire, and explosion are the most frequent causes of severe consequences. Such accidents are caused by misinterpretation of knowledge and communication issues.
(45, 121)	2013, 2018	Malaysia	A lack of training and guidance exposes people to dangerous chemicals because appropriate safety measures are not taken immediately. A lack of safety culture was identified as the leading cause of negligence and hazardous situations in organizations.
(129)	2020	Ghana	A lack of health and safety policies, untrained and underqualified employees, and poor infrastructure.
(52)	2022	China	Sets of safety performance factors that impact industries, including management and worker factors.
(53)	2020	Indonesia	Poor communication, the employees or people involved consider themselves fit for duty. Accidents occur due to a lack of information or knowledge, appropriate training, leader supervision, and implementation of occupational safety and health regulations.
(130)	2011	Iraq	Insufficient tools, poor technology, poor organizational management, lack of precautions, lack of adequate services, employee disregard of safety regulations, and inadequate training are factors that trigger unsafe behavior.
(131)	2019	Iran	Technological advances have excelled in high-risk involvements, the negligence of which forms the basis of catastrophe.
(74)	2019	UAE	In past studies, fatal accidents have occurred in the UAE oil and gas industry with different safety issues, including explosions, exposure to chemical equipment, and inspection of high-heating products (boiler), which are the leading causes of loss of workforce.

##### 3.5.2.3. Strategies

Safe operational practices are necessary for safe production at the workplace, primarily focusing on unsafe situations, where there is no established standard procedure for carrying out a specific task or where the legal mechanism is lacking for safely completing the task (132). Implementing a compelling communication vision in delivery systems might be achieved by an organization by establishing uniform standards and processes. Accident and injury rates can be reduced by establishing process checklists, standardizing care procedures, and removing pointless variance where practical (104). Standard mechanisms ensure that organizational tasks are completed safely and effectively and can contribute to a better and safer work environment (133). A control system must also be in place for an organization to operate safely. To ensure that these control systems are being used and are sufficient to handle the constantly changing working environment, they must be evaluated and monitored regularly. Their goal is to pinpoint the situations where a deficiency in the monitoring and reporting of present controls results in workplace hazards or injuries (132, 134).

##### 3.5.2.4. Safety rules and regulations

To protect the workers and safeguard the operational facilities of the oil and gas industry, several international and national organizations have enforced international and national rules to overcome health, safety, and environmental challenges. Accidents happen despite safety policies, rules and regulations, acts, laws, and training. In Malaysia, concerns related to oil and gas encouraged the enactment of the Petroleum Act of 1984 for transportation safety measures. The Act regulates the storage, transportation, and utilization of petroleum products. Another subsidiary regulation (Regulations of 1985) relating to petroleum safety measures was introduced for pipeline transport under the same Act (2, 135).

The Petroleum mining ACT was introduced in 1984 but had limited safety implications for offshore operations. To overcome the limitations and resolve safety concerns, another Act, namely the Factory and Machinery Act (FMA), was introduced in 1967, which provides the safety, health, and welfare governance of the workers along with the inspection and registration of machinery to ensure safe working environments. Therefore, offshore oil and gas platforms are subject to the FMA (135). Still, the ACT had certain shortcomings, which were fulfilled after introducing the Occupational Safety and health act (OSHA) in 1994. The OSHA promotes a safe and healthy work environment, including onshore and offshore installations. The OSHA ensures the safety of operations by mandating a safety committee that performs risk assessment and helps provide safety measures to prevent accidents and hazards (136, 137). Table 6 provides a brief overview of the different safety rules and regulations introduced by different countries to improve workplace safety.

**Table 6:** Brief overview of the different safety rules and regulations introduced by different countries to improve workplace safety.

Rules and regulations	Details	Country	Source
OSHA 1994	Mandates the establishment of a safety committee, employing safety officers, measuring chemical health risks at facilities with organizational incidents hazards, monitoring of organizational hygiene and medical surveillance of personnel, and all other safety mechanisms.	Malaysia	(136)
FMA 1967	Controls factories concerning matters relating to the safety, health, and welfare of persons therein.	Malaysia	(135)
ACT 1984	Consolidates the safety-related laws regarding transportation, storage, and utilization of petroleum and matters relating to it.	Malaysia	(2), (135)
Act 1974	Mandates the safeguarding of work-related health, safety, and welfare.	Malaysia and Singapore	(76)
2009 regulations	For operators considering safety cases, validation, and accident notification about offshore facilities.	Australia	(137)
Workplace Safety and Health Act (2009)	Provides a framework to protect all workers’ health, safety, and welfare concerning the workplace and work activities. International Labour Organization	Brunei	(131)
Act 2003/Law-11,970	Covers Indonesia’s paramount health and safety laws, focuses on their prevention, and ensures the implementation of safety measures.	Indonesia	(138)
Factory Act 1951 and OSHL 2019	Concerns the implementation of employee safety and health in the workplace.	Myanmar	(136, 139)
Republic Act (2006)	Established for occupational safety, health, and welfare.	Singapore	(140)
BSEE (2010)	The United States established the Bureau of Safety and Environmental Enforcement (BSEE) to respond to the Deepwater Horizon explosion in 2010; it strictly follows offshore safety and environmental protection rules and regulations.	United States	(141)

##### 3.5.2.5. Safety benchmark

The benchmarking process compares similar entities that need to improve in cost, quality, and performance. It refers to the idea of pursuing continuous performance improvement (CPI) by studying the top strategies used by competitors (142). A quality audit notifies auditors of performance gaps compared to the expected criteria. The benchmarks are often set based on performance targets, while the audit criteria are specified. Usually, surveys, audits, interviews, or industry standards are used to establish the measurements of the benchmarking process. Organizations identify performance gaps, select action items, and conduct follow-up studies to improve underperforming processes. Benchmarking strategies have been employed extensively in the O&G sector, just like in other industrial sectors.

##### 3.5.2.6. Safety indicator

When one or more leading indicators point to a missing or ineffective part of the safety program, interventions or adaptive mitigation measures can be implemented to strengthen the safety program and improve safety performance outcomes. The leading indicator measures a system, condition, or behavior that predicts future performance. In contrast, effective leading indicators should anticipate a change in performance, whether for the better or worse. A literature theoretical framework included three categories of safety performance indicators: result, monitor, and derive indicators. Result indicators measure the overall outcomes and conditions of a system like TRIR. Monitor indicators, such as work and safety motivation or hazard recognition, show the organization’s potential and capacity to conduct safety procedures. Derive indicators, such as safety leadership and strategic management, measure how well safety management operations are carried out. Using this theoretical framework, the authors stated that monitor and drive indicators are leading indicators. They control system activities and organizational dynamics, whereas outcome indicators lag because they result from various elements. The oil and gas industry usually gauges its safety performance using lagging indicators, such as the total recordable incident rate TRIR and the severity rate S.R. of the Occupational Safety and Health Administration (OSHA). Lagging indicators are metrics that relate to injury or loss events in the past. Lagging indicators highlight and respond promptly to hazards and accidents, and lagging indicators of safety performance are based on prior safety performance results (71, 143, 144). Table 7 assists in deciding the proactive metrics concerning their measurement, highlighting the leading and lagging indicators.

**Table 7:** Proactive metrics concerning their measurement, highlighting the leading and lagging indicators.

Proactive metrics	Measurements	Indicators
Safety training programs	Number of training sessions conducted. Percentage of employees trained. Number of safety violations pre- and post-training. TRAR and SR pre- and post-training.	Leading
Safety meetings	Frequency of safety meetings held. Participation rates of employees and management. Number of safety concerns raised and addressed. TRAR and SR pre- and post-meetings.	Leading
Job hazard analysis	Number of job hazard analyses completed. Number of corrective actions taken. Number of incidents prevented. TRAR and SR pre- and post-hazard analyses.	Lagging
Safety audits and inspection	Frequency of safety audits and inspections. Number of safety violations identified. Number of corrective actions taken. TRAR and SR pre- and post-audits/inspections.	Leading/ Lagging
Safety communication plans	Effectiveness of safety communication strategies. Employee feedback on safety communication. Number of safety incidents prevented. TRAR and SR pre- and post-communication plans.	Leading
Near-miss reporting system	Number of near misses reported. Percentage of near misses investigated and addressed. Number of incidents prevented through near-miss reporting. TRAR and SR pre- and post-near-miss reporting.	Leading/ Lagging
Management commitment to safety	Demonstrated commitment from upper management. Allocation of resources to safety initiatives. Employee perception of management commitment to safety. TRAR and SR pre- and post-commitment to safety.	Leading/ Lagging
Employee involvement in safety	Employee participation in safety programs. Employee feedback on safety policies and procedures. Employee suggestions for safety improvements. TRAR and SR pre- and post-employee involvement.	Lagging
Safety risk assessments	Number of risk assessments completed. Number of high-risk activities identified. Number of mitigating actions implemented. TRAR and SR pre- and post-risk assessments.	Lagging
Safety leadership	Demonstrated safety leadership from managers and supervisors. Employee perception of safety leadership. Number of safety initiatives led by safety leaders. TRAR and SR pre- and post-safety leadership.	Leading
Safety culture assessments	Employee perception of safety culture. Number of safety culture initiatives implemented. Number of safety culture improvements observed. TRAR and SR pre- and post-safety culture assessments.	Leading
Safety incentive programs	Employee participation in safety incentive programs. Number of safety-related achievements recognized. Number of safety incidents prevented through incentive programs. TRAR and SR pre- and post-incentive programs.	Leading
Safety checklists	Number of safety checklists completed. Number of safety issues identified. Number of corrective actions taken. TRAR and SR pre- and post-checklists	Lagging
Contractor management	Number of safety assessments completed for contractors. Number of safety requirements communicated to contractors. Number of safety incidents involving contractors. TRAR and SR pre- and post-contractor management.	Leading
Safety equipment inspections	Frequency of safety equipment inspections. Number of safety equipment issues identified. Number of corrective actions taken. TRAR and SR pre- and post-equipment inspections.	Lagging
Emergency preparedness	Effectiveness of emergency response plans. Number of emergency drills and exercises conducted. Number of emergency incidents prevented or mitigated. TRAR and SR pre- and post-emergency preparedness.	Leading
TRAR	Total Recordable Accident Rate (TRAR) is a safety performance indicator that measures the number of work-related accidents and injuries within a specific timeframe per a certain number of hours worked. It provides a standardized measure to assess the overall safety performance of an organization or workplace. TRAR = (Number of Recordable Accidents / Total Hours Worked) x 100,000	
Safety readiness (SR)	SR, or Safety Readiness, is a proactive safety measure that refers to the preparedness and state of readiness within an organization or individual to effectively manage safety risks and respond to potential hazards. It encompasses a range of activities and practices aimed at preventing accidents and promoting a safe working environment	
Pre- and post training	Pre- and Post-Training measures refer to proactive safety measures taken before and after training sessions to enhance the effectiveness of safety training programs and maximize their impact on improving workplace safety. These measures aim to ensure that employees receive relevant and comprehensive training, retain the knowledge and skills acquired, and apply them effectively in their work environment.	

#### 3.5.3. Safety performance

The rate of occupational injuries/accidents and hazards requires every worker’s participation. Commitment to unsafe Acts and unsafe conditions could enhance workplace safety if every individual adhered to the organizational safety rules and was eager to strengthen safety performance (57). Similarly, (121) revealed positive safety performance-maintained safety measures, procedures, and rules derived from regular communication between supervisors and employees. Some accidents are impossible to prevent in high-risk working environments due to the nature of certain operations and processes. Though industries are now strictly adhering to safety precautions for health, safety, and the environment, incidents are still reported worldwide, to which the oil and gas industry contributes the majority. According to worldwide evidence, 2 million fatalities are reported relating to accidents and diseases caused by the workplace. A total of 270 and 160 million occupational accidents and illnesses occur annually (28, 29). Workplace safety remains challenging and it is necessary to avoid accidents effectively and improve safety performances (38).

##### 3.5.3.1. Occupational accidents

Occupational accidents result in injuries requiring medical attention (145). Reducing occupational accidents is perceived as the goal or outcome of an organization’s safety efforts. Occupational accidents result from many factors, including unsafe behavior, (which is a direct trigger factor, with it intends to highlight different level of injuries) in most organizational injury rates (124). Occupational accidents can also be measured by recordable injuries resulting in lost time, recordable injuries requiring medical treatment, and incident rates based on severity and frequency (60). This is vital in a high-risk work environment, specifically in the oil and gas industry, where disasters and accidents can occur if the information is not communicated adequately among workers. Negligence and information blocks could result in disasters and accidents.

##### 3.5.3.2. Occupational fatalities accidents

ILO global statistics report nearly 2.78 million annual fatalities. Poor and inefficient safety systems, management practices, human error, etc. were the causes of the reported incidents. Such consequences are alarming, and improving the workplace to reduce workplace accidents, injuries, and fatalities is necessary. Furthermore, such safety issues in the workplace need to be identified and addressed appropriately to mitigate risks in the workplace because they also accrue costs to the organization in terms of medical, health, death, incapacitation, equipment damage, production loss, and increased insurance costs (9–12). The fatality rate due to workplace accidents among oil and gas employees is seven (7) times higher than in other industries. As a result, companies in this sector must provide a safe working environment to reduce the risks of operational hazards.

##### 3.5.3.3. Lost time injury

There are two methods for reporting lost time: lost time injuries (the subset of work-related injuries that result in ‘lost time’ due to work absence) and lost time injury frequency rate [the number of lost time work-related injuries (fatalities and failed workday cases) per one million work hours (146, 147)]. Some companies, however, calculate lost time injury frequency rates according to the Occupational Health and Safety Administration guidelines, which use 200,000 h as the denominator (147).

##### 3.5.3.4. Near misses

Analyzing the literature has revealed two main approaches to a near-miss definition. The first (traditional approach) considers a near miss to be any event that occurs but does not cause any harm. Sometimes, it may only cause minor property damage. The second (proactive approach) includes events and potentially hazardous situations, such as unsafe acts and conditions. Some recent studies have outlined the main features of the first approach by analyzing it. In a review, the concept of precursors was critically examined (80), who attempted to clarify the definition of a near miss by analyzing a generic accident sequence. A near miss is defined in this framework as a precursor to an accident in which the accident sequence has been prevented, although the accident could have taken place.

#### 3.5.4. Safety commitment

Safety commitment is a crucial component of safety practice to ensure that safety in the organization is successfully achieved, especially in the oil and gas industries, which face high incident rates. Thus, safety commitment is the involvement of an individual in safety activities to improve workplace safety and can reduce the costs to organizations and any loss of workforce (77, 148, 149). Numerous studies have found that if organizations follow safety rules and procedures and conduct regular safety training, and if management shows commitment, attention, and interest in safety issues, they are likely to have a lower rate of injuries and accidents (99, 150, 151). Various researchers (152, 153) have highlighted that safety rules and feedback have a positive relationship with safety management related to management’s commitment to safety in the workplace. According to (121, 154), fostering workplace safety and executing safety practices in the workplace is difficult without management commitment to safety, which shows that the management of safety is an essential element in ensuring effective and efficient safety practices in an organization. A total of 13 previous studies (32%) highlighted the characteristics related to safety motivation, specifically in high-risk work environments. Most of the authors focused on and discussed the sub-theme of safety participation, which was the focus of 15 articles (38%), followed by safety knowledge, as the focus of 14 articles (35%), and safety compliance, as the focus of 13 articles (32%).

##### 3.5.4.1. Safety participation

Safety participation is a system that develops an overall participant environment, although it does not directly affect an individual’s safety (41, 155). A safe work environment requires participation in safety committees, reporting on unsafe situations, decision-making, suggestions for enhancing security, and motivating guide co-workers involved in dangerous acts. Safety participation refers to supporting employee behavior and creating an environment to support safety as well as procedures for helping co-workers, involvement in voluntary safety activities, taking safety initiatives, promoting safety-related principles, and implementing safety in the workplace.

##### 3.5.4.2. Safety knowledge

Safety knowledge is based on the degree of workers’ knowledge regarding the organization’s safety system, practices, and procedures. Employee work performance is determined according to their understanding, motivation, and skill; thus, workers perform their duties safely by following safety measures to enhance these three factors. Organizations must conduct frequent, comprehensive, and systematic health and safety training programs to improve employees’ knowledge (129). Workers’ knowledge related to performing their jobs safely, how to use safety equipment and standard operating procedures, how to maintain or improve safety and health in the workplace, how to reduce the risk of accidents and incidents in the workplace, the associated hazards and necessary precautions, and how to report potential hazards noticed in the workplace are the six items of the safety knowledge scale (156).

##### 3.5.4.3. Safety compliance

A significant relationship between accident involvement and unsafe behavior is understood to be based on actions, participation in risks, violating rules, and unsafe behavior. These create a highly hazardous situation for an individual, eventually leading to critical accidental events. Lack of safety knowledge leads to safety rules being ignored and failure to use appropriate protective personal equipment. The previously mentioned examples are called safety compliance, which develops only one aspect of safe behavior (157, 158). Safety compliance implies workers’ behavior in relation to safety procedures and policies for achieving work safety standards, such as obeying rules regarding the use of personal equipment, performing operational duties safely, complying with safety rules and regulations, and following appropriate procedures (8). The oil and gas industry is highly regulated with safety standards and procedures. Occupational safety rules within the oil and gas industry completely rely on safety, and virtually operational tasks are processed by rules and regulations. Therefore, a high level of compliance requires a high level of safety preconditions.

##### 3.5.4.4. Safety motivation

Safety motivation, or the desire to participate in safety practice that will enhance one’s skills and improve safety behavior, is another proximal antecedent of safety performance (145, 156). The necessity of maintaining safe and effective practices, making an effort to improve personal safety, and reducing the risk of workplace accidents and incidents are used as a scale to measure safety motivation (107). The safety climate was found to be significantly correlated with safety knowledge and motivation in meta-analysis studies, both of which are associated with predicting safety performance, which indirectly affects accidents and injuries (145, 159). Improvements in information and training that promote safe behavior can improve worker health and safety (88, 160).

### 3.6. Main findings

To summarize, the findings of this study provide valuable insights into the themes of safety communication, occupational health and safety, safety performance, and safety commitment within high-risk industries, particularly the oil and gas sector. These themes encompass 18 sub-themes that were identified through an extensive analysis of the literature. The major findings and their implications are discussed as follows.

Safety communication emerged as a critical factor in ensuring the well-being of workers in high-risk organizations. Effective safety communication practices were found to enhance the efficiency of the safety management system and guide workers in reducing unsafe incidents. A lack of safety communication was identified as a significant factor in conflict and confusion among workers regarding safety rules and procedures. The sub-themes of communication climate, effective communication, communication mechanism, and communication satisfaction were highlighted in the literature, emphasizing their relevance in organizational safety performance. The presence of a supportive communication climate, characterized by open and two-way communication, was found to promote symmetrical communication within the organization. Transformational leadership behavior was identified as a key influencer of workers’ perspectives on communication climate, highlighting the importance of leadership in fostering effective communication. It can be concluded that symmetrical internal communication contributes to achieving organizational goals and establishing a strong relationship between employees and the organization.

Occupational health and safety management was identified as a crucial aspect of maintaining workplace safety and preventing hazards and injuries. Safety policies, safety issues, safety strategies, safety rules and regulations, safety benchmarking, and safety indicators were among the sub-themes explored in this area. Safety policies were found to play a vital role in demonstrating management’s commitment to safety and in setting clear guidelines and procedures. Safety issues, including hazards and injuries, were identified as ongoing challenges in the oil and gas industry, indicating the need for continued efforts to address these concerns. Strategies such as process standardization and control systems were emphasized as essential for maintaining a safe working environment. Compliance with safety rules and regulations was identified as a critical factor in protecting workers and preventing accidents. Safety benchmarking and safety indicators were recognized as valuable tools for monitoring and improving safety performance.

Safety performance, including occupational accidents, occupational fatalities, lost time injuries, and near misses, was a key area of focus in the literature. The study revealed the importance of understanding the factors contributing to occupational accidents and the need for effective safety measures to prevent such incidents. Occupational fatalities and lost time injuries were identified as significant concerns, emphasizing the need for comprehensive safety systems and management commitment. The concept of near misses was explored, highlighting its potential as a proactive indicator of potential accidents and the importance of reporting and addressing near misses to prevent future incidents.

Safety commitment was identified as a critical component of safety practice and performance. The sub-themes of safety participation, safety knowledge, safety compliance, and safety motivation were examined in this context. Active participation in safety activities, knowledge of safety practices and procedures, compliance with safety rules, and motivation to prioritize safety were all found to contribute to a positive safety culture and improved safety outcomes. Management commitment to safety was identified as a crucial factor in fostering a safe working environment and encouraging employee involvement in safety practices.

Overall, this study provides comprehensive insights into the themes of safety communication, occupational health and safety, safety performance, and safety commitment within high-risk industries. The findings underscore the importance of effective communication, robust safety management systems, and a strong safety culture in ensuring the well-being of workers and preventing accidents and injuries. The identified sub-themes offer valuable avenues for further research and provide practical implications for organizations seeking to enhance their safety practices and performance in high-risk workplaces.

## 4. Discussion

The purpose of this study was to conduct a comprehensive literature review on the impact of organizational variables on effective safety communication, communication climate, communication satisfaction, and commitment to safety. The findings of the reviewed articles revealed that organizations are influenced by various factors in their pursuit of creating a healthy and safe work environment. The study considered two databases and identified 40 articles relevant to the impact of effective safety communication. From the extracted literature, the study identified four main themes, namely, occupational health and safety, safety communication, safety performance, and safety commitment, which collectively contribute to achieving the organizational goals and visions of a safe workplace.

Safety communication was highlighted as a critical aspect of safety management systems in the oil and gas industry. It involves the exchange of information among workers, supervisors, managers, and stakeholders regarding safety policies, procedures, and regulations. Different forms of safety communication were identified, such as safety meetings, alerts, training sessions, reports, audits, and feedback mechanisms. Effective safety communication enables workers to identify and report hazards, take corrective actions, and learn from safety incidents, ultimately preventing future occurrences (28, 50, 57, 161, 162).

The communication climate within an organization plays a crucial role in safety communication. A positive communication climate, characterized by open and honest communication, mutual respect, and trust, fosters effective safety communication. In such an environment, workers are more likely to speak up about safety concerns, ask questions, and offer suggestions for improving safety practices. Establishing a positive communication climate encourages collaboration and problem-solving among workers, leading to enhanced safety outcomes (41, 93, 97).

Communication satisfaction also emerged as a significant factor in safety communication. When workers feel satisfied with the communication they receive from supervisors, colleagues, and stakeholders, they are more likely to engage in safety communication. Satisfied workers are more inclined to report safety incidents and hazards, offer suggestions for improvement, and adhere to safety procedures and regulations. Therefore, organizations should strive to ensure that workers receive clear, timely, and supportive communication, which positively impacts safety performance (63, 67, 113, 163, 164).

To facilitate effective safety communication, the study highlighted the importance of having an appropriate communication mechanism in place. Workers need access to tools and channels that enable them to report incidents, receive safety information, and communicate with colleagues and supervisors. Various mechanisms were identified, including safety manuals, meetings, committees, hotlines, suggestion boxes, training, and inspections. Employing the right communication mechanisms enhances safety communication and improves overall safety performance (63, 70, 164, 165).

Communication effectiveness (C.E) in safety communication refers to the degree to which safety information is communicated accurately, clearly, and in a timely manner to all relevant stakeholders in the oil and gas industry. Effective safety communication ensures that workers are aware of safety hazards, understand safety procedures and regulations, and can take appropriate measures to prevent accidents and incidents. Communication effectiveness is crucial to the success of safety communication in the oil and gas industry. Effective safety communication ensures that safety information is accurately and clearly communicated to all relevant stakeholders, enabling workers to understand safety procedures and regulations, identify safety hazards, and take appropriate measures to prevent accidents and incidents (72, 113, 117, 163, 166, 167).

Occupational health and safety (OHS) management systems were found to be crucial in creating a safe work environment. These systems encompass the development and implementation of rules, regulations, and policies that promote workplace health and safety. Effective safety policies motivate employees and managers to perform their duties in accordance with safety procedures, reducing the occurrence of unsafe conditions and acts. Organizations should allocate appropriate resources to ensure the successful implementation of safety measures and prioritize safety within their budget evaluations (49, 168–176).

Safety commitment, as a subset of safety management strategies, was identified as a key factor in influencing safety performance. Top management plays a vital role in motivating and influencing workers’ safety performance through their commitment to safety. Transformational leadership was highlighted as an effective leadership style that influences safe employee behavior, contributing to high safety performance. Organizational support, open communication climates, and management involvement in decision-making processes all contribute to a supportive safety environment that fosters safety participation and compliance (177–182).

Safety performance indicators, including occupational accidents, lost time injuries, fatalities, near misses, and occupational diseases, provide measurable insights into organizational safety performance. The severity of an occupational accident is determined by its impact on property damage and injuries. Analyzing incidents helps uncover underlying factors, such as deficiencies in OHS activities and programs, and individual unsafe behaviors that trigger accidents. Organizations should monitor leading safety indicators and utilize appropriate tools and technology to identify and address safety issues proactively (145, 183).

To conclude, this comprehensive literature review emphasizes the significance of organizational variables in effective safety communication and the creation of a healthy and safe work environment. By understanding and addressing factors such as communication climate, communication satisfaction, communication mechanisms, and safety commitment, organizations can improve safety performance and prevent accidents and injuries.

### 4.1. Practical relevance

The findings of this study hold significant practical relevance for safety practitioners operating in high-risk workplace environments. By highlighting the importance of safety communication, occupational health and safety management, safety performance, and safety commitment, the study provides valuable insights that can inform and guide the practices of safety professionals.

Firstly, the emphasis on safety communication underscores the need for safety practitioners to prioritize effective communication strategies within their organizations. By promoting a supportive communication climate, implementing effective communication practices, establishing clear communication mechanisms, and fostering communication satisfaction, safety practitioners can enhance the flow of safety-related information, reduce conflicts and confusion, and ultimately improve safety outcomes. The study’s findings provide guidance on key areas of focus for safety communication initiatives, enabling practitioners to develop targeted and impactful communication strategies that address the specific needs of their organizations.

Secondly, the exploration of occupational health and safety management highlights the importance of implementing comprehensive safety policies, addressing safety issues, devising effective safety strategies, ensuring compliance with safety rules and regulations, benchmarking safety performance, and utilizing safety indicators. Safety practitioners can utilize these findings to assess and strengthen their organization’s safety management systems. By adopting standardized safety policies, identifying and mitigating safety issues, implementing effective safety strategies, and staying up to date with relevant safety regulations, practitioners can contribute to creating a safer working environment. Furthermore, the study’s insights on safety benchmarking and safety indicators offer practical tools for safety practitioners to monitor and evaluate safety performance, identify areas for improvement, and drive continuous safety enhancement initiatives.

Moreover, the study’s examination of safety performance provides safety practitioners with valuable information on occupational accidents, fatalities, lost time injuries, and near misses. By understanding the factors contributing to these incidents, safety practitioners can develop targeted prevention strategies, improve safety training and procedures, and implement proactive measures to identify and address potential hazards. The findings emphasize the importance of management commitment, employee participation, safety knowledge, and safety motivation in achieving positive safety performance. Safety practitioners can leverage these insights to design and implement comprehensive safety programs, enhance safety training initiatives, and foster a safety culture that encourages active involvement and prioritization of safety.

Lastly, the study’s focus on safety commitment underscores the critical role of management in creating a safe working environment. Safety practitioners can use this knowledge to advocate management support and commitment to safety initiatives, ensuring that safety practices and procedures are effectively implemented and adhered to throughout the organization. By promoting safety participation, enhancing safety knowledge, fostering safety compliance, and encouraging safety motivation, safety practitioners can drive a culture of safety commitment among employees, leading to improved safety performance and reduced risks.

## 5. Limitations and challenges

This study is based on thematic analysis results; the selected review articles on the practice of safety communication in the high-risk workplace is still moderate and has only increased in recent years. Most papers are published in the construction industry rather than other high-risk industries, such as oil and gas, mining, and manufacturing. Furthermore, most of the articles were removed during the screening process because the focus area was the oil and gas industry, indicating a gap and the need for further investigation and research in these industries. Since OHS reporting methods and procedures depend on industries and the nature of work, further research is required to focus on and identify OHS performance measures and indicators that are more promising and can assist an organization in effectively implementing them (183). Future research is required in a wide range of oil and gas industries, which may have more OHS procedures and standards due to the use of benchmarking tools or adapting across safety communication activities.

Based on the current research, most of the selected review papers focused on occupational accidents and injuries. All were supported by (124), who identified that most organizations provide detailed information about occupational injuries. Thus, focus should be applied to all levels and types of accidents, despite the level of loss or damage, such as occupational accidents, fatalities, near misses, and lost time injuries. Minor accidents that do not have severe outcomes or that do not damage property and equipment still require attention as they can cause future incidents. Future research should consider a lack of effective communication, safety communication, and commitment towards safety as safety outcomes as the root causes of fatal occupational accidents, occupational accidents, lost time injuries, and near misses. Thus, the researcher should also highlight the positive outcomes of high safety standards in the workplace, such as economic increase, productivity, and profit increase.

Despite the abovementioned limitations, the results of this systematic review could draw a framework relevant to developing strategies regarding effective safety communication and safety commitment research and to organizations seeking to improve the safety behavior of workers in the oil and gas industry. These findings are also relevant to other high-risk industries, where safety procedures, rules, and regulations constitute a major concern for maintaining safety.

## 6. Conclusion

This study is based on a systematic review, emphasizing the importance of the variables that influence the reduction of accident and injury rates through effective communication and commitment to safety. In an organizational context, the minimum rate of incidents vision focused on the occupational health and safety environment. Most organizations and policymakers implement this vision efficiently along with strategies and programs related to occupational health and safety. Various scholars have experiences associated with OHS from different backgrounds, giving insight into its effective use and success from a theoretical perspective and in practice. The countries where the highest number of studies were conducted were Malaysia, with seven articles (18%); Nigeria, with three articles (8%); and Norway, with three articles (8%), followed by China, Iran, the U.S., Australia, Bahrain, and Indonesia, each with two articles (5%). Most of the countries published one article only, including Canada, Brazil, Italy, the UK, Saudi Arabia, France, Egypt, Brunei, Albania, Israel, Croatia, Columbia, Singapore, and the UAE.

This study’s systematic review emphasizes the importance of the variables that influence the reduction of accident and injury rates through effective communication and safety commitment. The findings shed light on various dimensions of safety communication, occupational health and safety management, safety performance, and safety commitment, providing valuable insights for both the industrial and academic realms.

One significant implication of this study is the development of strategies to promote a safety culture and address communication barriers. The findings highlight the need for interventions that enhance communication skills and techniques at both macro and micro levels. Safety practitioners can leverage these insights to design communication management strategies that foster a culture of safety and overcome obstacles in safety communication.

Moreover, this study also reveals several gaps and potential areas for future research. One such area is the exploration of safe behavior as an influencing variable on safety principles. Future studies could conduct in-depth qualitative analyses to provide a comprehensive understanding of safe behavior and its impact on organizational safety. Additionally, future research could employ specific systematic review methodologies, incorporating appropriate searching techniques such as reference searching, citation tracking, snowballing, and expert input, to further enhance the research synthesis in this field.

Furthermore, the study highlights the need to adapt to new challenges and hazards in the context of the 4th Industrial Revolution and the global impact of events like the COVID-19 pandemic. Future studies should focus on developing new strategies that ensure the sustainability of safety in the face of emerging challenges. This includes strengthening safety competencies among designers, engineers, and workers to maintain a safe working environment.

Practitioners can derive practical leads from this study to improve organizational commitment to safety, devise strategies to overcome communication barriers, foster a safety culture, and enhance communication skills and techniques. The comprehensive overview of the elements and their relationship with negative safety outcomes and performance provides practitioners with valuable insights for developing and evaluating interventions aimed at improving occupational safety.

In conclusion, this study not only presents significant findings on the importance of communication and safety commitment in occupational safety but also offers valuable directions for future research. By focusing on the development of effective communication strategies for safety commitment and addressing the identified research gaps, researchers can contribute to advancing the field and promoting a safer and healthier working environment.

## Data availability statement

The original contributions presented in the study are included in the article/supplementary material, further inquiries can be directed to the corresponding author.

## Author contributions

JZ, SN, and AI contributed to the design and implementation of the study. JZ conducted the detailed analysis and manuscript drafting. SN contributed to assessing the study quality and management of the study. SN and AI were involved in supervision and manuscript revisions. All authors contributed to the article and approved the submitted version.

## Funding

The research was supported by Centre for Graduate Studies (CGS), Universiti Teknologi PETRONAS, and Yayasan Universiti Teknologi PETRONAS (YUTP) through the grant cost center YUTP 015LC0-374.

## Conflict of interest

The authors declare that the research was conducted in the absence of any commercial or financial relationships that could be construed as a potential conflict of interest.

## Publisher’s note

All claims expressed in this article are solely those of the authors and do not necessarily represent those of their affiliated organizations, or those of the publisher, the editors and the reviewers. Any product that may be evaluated in this article, or claim that may be made by its manufacturer, is not guaranteed or endorsed by the publisher.
